# Prediction of isometric forces from combined epidural spinal cord and neuromuscular electrical stimulation in the rat lower limb

**DOI:** 10.21203/rs.3.rs-3377679/v1

**Published:** 2023-10-04

**Authors:** Daniel Song, Matthew Tresch

**Affiliations:** Northwestern University; Northwestern University

## Abstract

Both epidural spinal cord and muscle stimulation have been widely used for restoration of movement after spinal cord injury. However, using both approaches simultaneously could provide more flexible control compared to using either approach alone. We evaluate whether responses evoked by combined spinal and muscle stimulation can be predicted by the linear summation of responses produced by each individually. Should this be true, it would simplify the prediction of co-stimulation responses and the development of control schemes for spinal cord injury rehabilitation. In anesthetized rats, we measured hindlimb isometric forces in response to spinal and muscle stimulation across a range of amplitudes. Force prediction errors were calculated as the difference between predicted co-stimulation vectors and observed co-stimulation vectors whereby small errors signified evidence for linear summation. We found that the errors for spinal and muscle co-stimulation were significantly larger than expected. Using a bootstrapping analysis, we find that these larger errors do not reflect a nonlinear interaction between spinal and muscle responses. Instead, they can be attributed to the variability of spinal stimulation responses. We discuss the implications of these results to the use of combined muscle and spinal stimulation for the restoration of movement following spinal cord injury.

## Introduction

Functional electrical stimulation (FES) is a useful approach for producing movements as well as facilitating recovery of voluntary movement after spinal cord injury (SCI) ^1,2^. One stimulation modality, epidural spinal cord stimulation, has been widely used to evoke and modulate muscle activity in patients with SCI ^3–6^. Such activity is produced by recruitment of local spinal networks and so can be evoked even in the absence of supraspinal input in cases of complete SCI ^6–8^. Epidural spinal cord stimulation has also been used to facilitate voluntary movements in people with incomplete SCI, modulating spinal networks to facilitate otherwise ineffective movements produced by residual descending systems ^9–11^. This has made spinal stimulation a promising approach to restore standing and stepping function after SCI ^12^. Another approach for restoring functional movements after SCI is neuromuscular electrical stimulation ^13^. This approach can be used to produce stepping patterns and other functional movements by sequentially activating individual muscles and fine-tuning stimulation parameters ^14–16^.

While each modality has been used in research and clinical applications, a combined spinal and muscle co-stimulation approach might work synergistically to provide a variety of benefits not afforded by either modality separately. Spinal stimulation is capable of evoking basic functional movements, such as limb extension or flexion ^17^, but due to the overlap of motor pools along the spinal cord, it can be difficult to fine tune these movements to adapt to specific task demands. Muscle stimulation, on the other hand, can activate muscles individually, thereby enabling more granular control to achieve task demands. While it is theoretically possible to implant many muscles for stimulation, specifying and controlling many muscles to ensure effective behavior is challenging and the extensive implants can be susceptible to hardware failures ^18^. By stimulating a select few muscles to adjust responses evoked by spinal stimulation according to specific task requirements, an approach that combines both modalities might improve the functionality of evoked movements without substantially increasing the complexity of control. As day-to-day function requires adaptation to a variety of perturbations and task requirements, the flexibility afforded by such an approach might help to improve the quality of life for people with SCI.

In order for such a spinal and muscle co-stimulation approach to be effective for restoring function, the responses from spinal and muscle stimulation should ideally combine simply and predictably. However, there are many potential ways that the effects of combined spinal and muscle stimulation might be difficult to predict. These include non-linear interactions due to collision of action potentials ^19^ in either afferent or efferent peripheral axons producing smaller than expected responses. Another possibility is non-linear synaptic integration and processing of sensory inputs in the spinal cord that may produce larger than expected responses. By making the responses evoked from combined stimulation difficult to predict, such factors would increase the complexity of producing precisely controlled movements using combined muscle and spinal stimulation.

Alternatively, if the responses from spinal and muscle stimulation combined linearly, it would greatly simplify the restoration of movements by combined stimulation. Previous work using intraspinal stimulation showed that combined stimulation of two different sites in the spinal cord can usually be predicted by the linear vectorial summation of each site’s response individually ^20–22^. Additionally, previous work using muscle stimulation demonstrated that the isometric force produced by simultaneous stimulation of two muscles could be predicted by the linear summation of forces produced by each muscle individually ^23^. It is unknown, however, if this property holds true for combined epidural spinal cord and muscle stimulation and we assess this possibility in our study.

## Results

### Spinal and muscle stimulation responses

**Figure 1:**
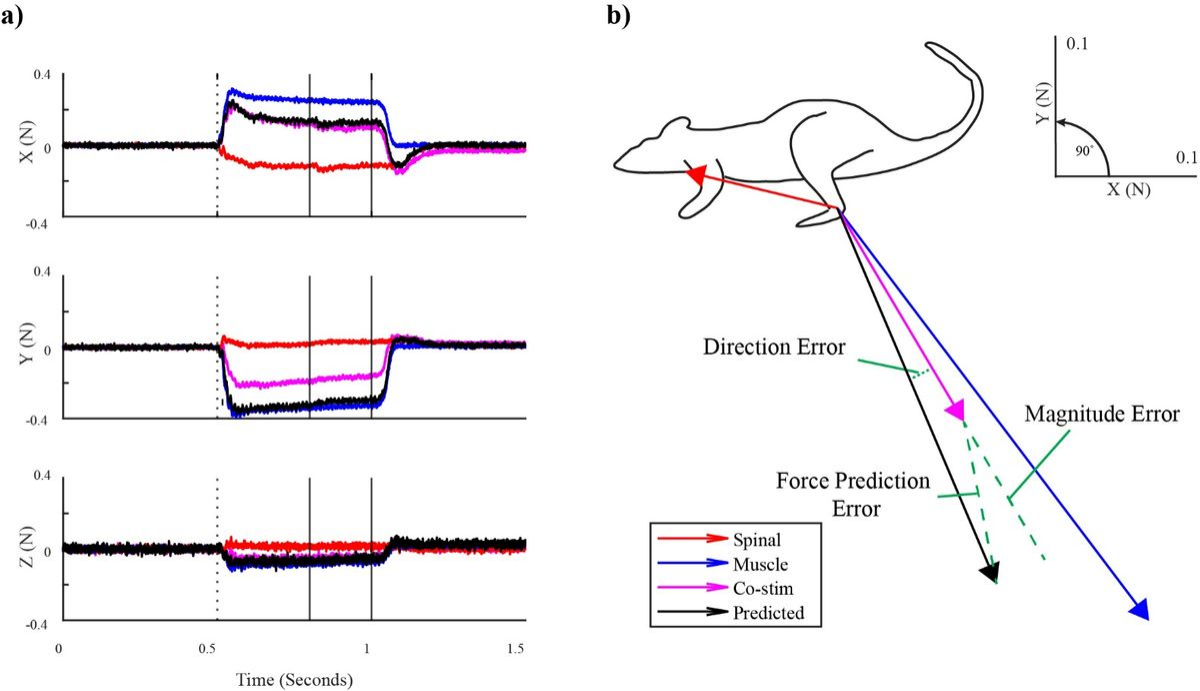
Example stimulation trials using a spinal stimulation amplitude of 222 µA at S1 and muscle stimulation amplitude of 500 µA at the vastus lateralis muscle. **a)** Force recordings in response to stimulation. Dotted vertical lines indicate the stimulation onset. Solid black lines indicate the time period used to calculate the isometric forces. **b)**Sagittal plane view of isometric force vectors from responses in a) showing visual representations of the different errors used to compare predicted and observed co-stimulation forces.

**Figure 2:**
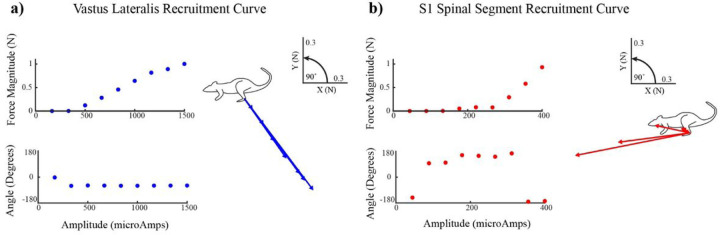
Example recruitment curves for stimulation at muscle and spinal sites illustrating how the magnitude (top) and direction (bottom) of the evoked responses changes with stimulation strength. The corresponding vectors are shown on the right of each recruitment curve. **a)**Recruitment curve in the Vastus Lateralis muscle. **b)** Recruitment curve in the S1 spinal segment.

Figure 1 shows an example of spinal/muscle stimulation trials at a relatively low amplitude. The forces peaked shortly after stimulation onset and leveled out thereafter in the latter portion of the stimulation train producing a smooth isometric force (Fig. 1a). The muscle stimulation produced limb extension, whereas the spinal response produced a rostral flexion. A prediction vector was calculated using the linear summation of the individual muscle and spinal vectors which was used to calculate error values for assessing linearity (Fig. 1b; see next section). Figure 2 shows recruitment curves from stimulation of the same muscle and spinal sites as in Fig. 1 (Muscle: VL, Fig. 2a; Spinal: S1 Fig. 2b). Increasing the stimulation amplitude applied to the muscle caused a monotonic increase in evoked force magnitude with minimal change in force direction (Fig. 2a). Increasing the stimulation amplitude applied to the spinal site also caused a monotonic increase in evoked force magnitude, but the direction of the evoked forces changed considerably as stimulation strength increased. For low spinal stimulation amplitudes in 6/6 animals, responses were directed rostral and dorsal, driving the limb towards flexion. As stimulation amplitude increased, in 4/6 animals force directions rotated and became directed ventral and caudal, driving the limb towards extension; in the remaining 2 animals, evoked forces remained directed in flexion, though some rotation did occur (Fig. 2b). Because of this change in direction with increasing stimulation strength, the range of force directions observed in individual spinal recruitment curves was considerably larger than those of muscle recruitment curves (angular deviation of 47.6°+/−20.9° for spinal vs. 2.7°+/−2.0° for muscle recruitment curves, Welch’s t test p < .05).

### Evaluating linearity of spinal/muscle co-stimulation

**Figure 3:**
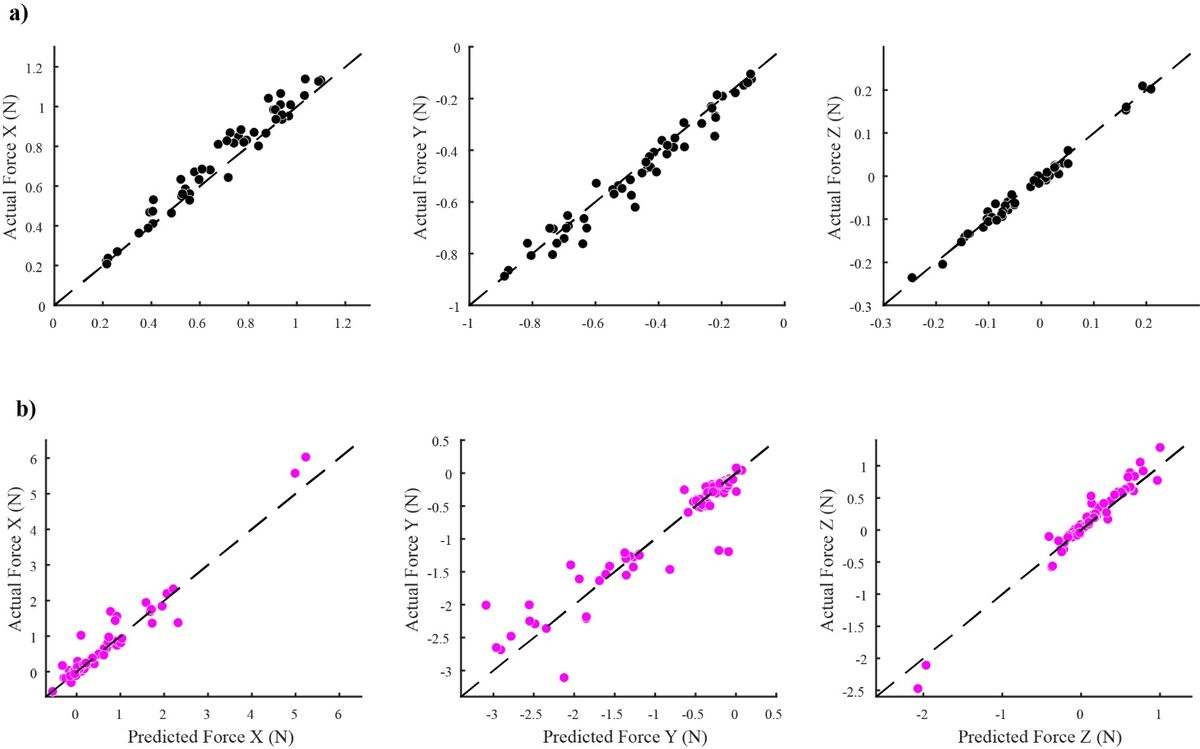
Linearity of co-stimulation assessed by combined R^2^ values which was calculated by taking the combined squared sum of residuals of each axis and divided by the sum of all total sum of squares each axis (i.e. Each axis had its own total sum of squares). The dashed line indicates perfect prediction. **a)** Linearity of muscle co-stimulation (Vastus Lateralis and Biceps Femoris). The predicted and actual forces match closely for all axes (combined R^2^ = .95). **b)** Spinal/muscle predicted forces also line up well with actual forces (combined R^2^ = .92) but not as well as muscle co-stimulation.

A total of 27 unique cases of muscle/muscle co-stimulation (n = 3,6,4,6,4,4 in each animal) amplitudes and 35 unique cases of spinal/muscle co-stimulation amplitudes were collected (n = 6,4,5,10,4,6 in each animal). Some cases were repeated more than once since there were multiple blocks of stimulation per rat. As expected, the forces produced by muscle/muscle co-stimulation were very similar to the forces predicted from the linear sum of the forces evoked by stimulation of each muscle individually (R^2^ = 0.95, Fig. 3a). Figure 3b shows the corresponding results for the spinal/muscle cases. Although the forces produced by spinal/muscle co-stimulation were also generally similar to the forces predicted from the linear sum of individual muscle and spinal sites (R^2^ = 0.92), the similarity was lower than that observed for muscle/muscle co-stimulation.

**Figure 4:**
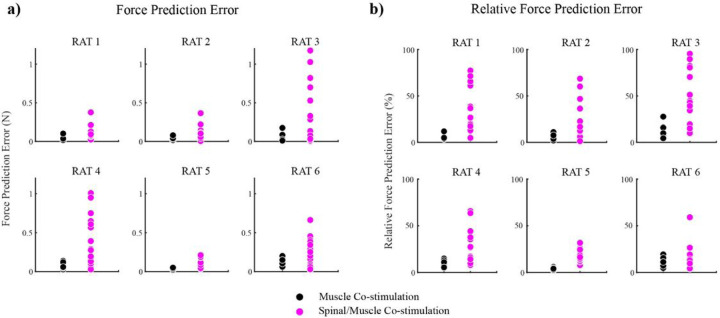
**a)** Force prediction errors and **b)**relative force prediction errors for each rat. Each point represents the error for a pair of spinal and muscle amplitudes. Some amplitude pairs were repeated more than once. Across all rats, spinal/muscle co-stimulation force prediction errors are larger than muscle co-stimulation pairs.

To evaluate the linearity of spinal/muscle co-stimulation quantitatively and compare it to the linearity of muscle-muscle co-stimulation, we calculated the Euclidean distance between the observed co-stimulation force vectors and the predicted co-stimulation force vectors (force prediction error, See Fig. 1b). Figure 4a summarizes the force prediction errors for muscle/muscle and spinal/muscle cases for each animal. Each data point represents the error for a combination of stimulation amplitudes. The median force prediction error across all subjects and trials was .11 N (error relative to co-stim magnitude: 18%) for spinal/muscle co-stimulation and .06 N (error relative to co-stim magnitude: 7%). Using a linear mixed model with subject as random factor and co-stimulation type (spinal/muscle or muscle/muscle) as fixed factor, force prediction errors were found to be significantly larger for spinal/muscle cases compared to muscle/muscle cases (p < 0.001). Both direction and magnitude errors were significantly larger for spinal/muscle cases (p < .05 for both).

**Figure 5:**
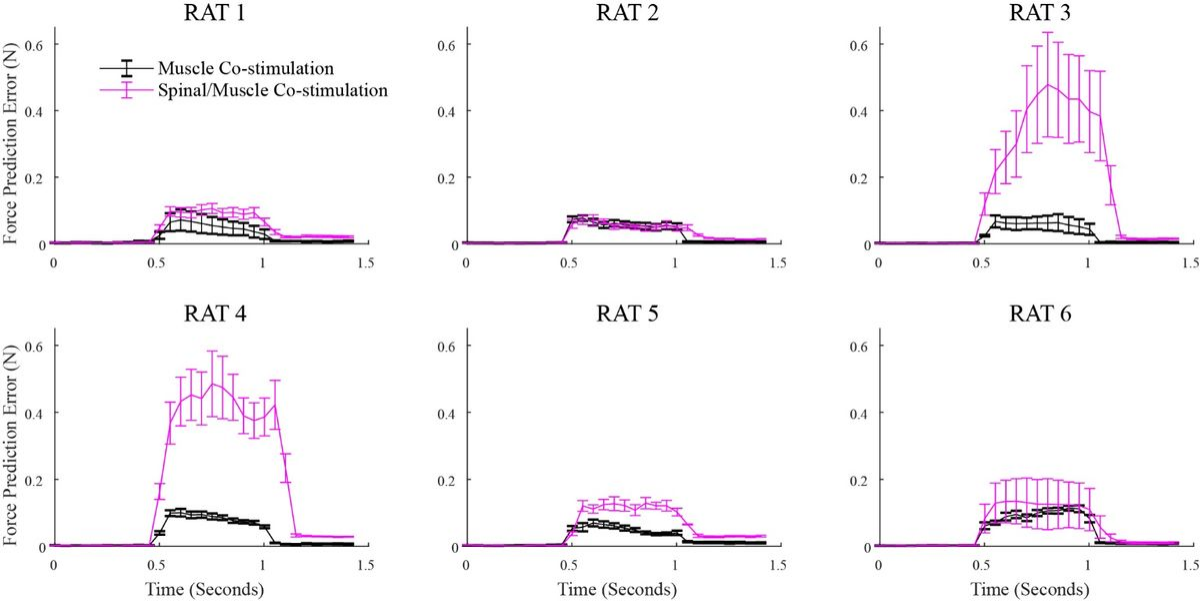
Force prediction errors across the stimulation duration for both spinal/muscle and muscle co-stimulation trials. Each co-stimulation trial was separated into 50 ms time windows. A force prediction error was then calculated for each time window. Error bars represent the standard error of the mean across co-stimulation trials within each rat.

We also evaluated whether force prediction errors varied across the duration of the co-stimulation train, potentially reflecting dynamics of spinal processing. Figure 5 shows the prediction errors for muscle/muscle and spinal/muscle co-stimulation cases at distinct time points in the stimulation train for each animal. There was no obvious relationship between the size of the prediction error and when in the stimulation train the error was calculated. To evaluate this possibility further, we considered only spinal/muscle co-stimulation trials and compared prediction errors between early (time window: 100 ms – 300 ms) or late (time window: 300 ms – 500 ms) in the stimulation train. We found no difference in error between these two periods of time in the stimulation train when used as a fixed factor in the mixed model analysis (p > .05).

### Potential sources for larger spinal/muscle co-stimulation prediction errors

We next examined potential reasons for the observed larger prediction errors for spinal-muscle co-stimulation trials. One possibility is that force prediction errors for spinal/muscle cases were higher simply due to differences in the magnitude of the co-stimulation force vectors ^23^, since spinal/muscle co-stimulation magnitudes were on average larger than muscle/muscle co-stimulation magnitudes (mean spinal/muscle co-stimulation magnitude: .97 N; mean muscle/muscle co-stimulation magnitude: .87 N). If error scaled with evoked magnitude^23^, this difference might explain the observed larger prediction errors for spinal/muscle co-stimulation. We addressed this possibility in two ways. First, the linear mixed model was rerun including co-stimulation force magnitude as a fixed effect, ‘nuisance’ variable. Even after doing so, the difference between spinal/muscle and muscle/muscle prediction errors remained significant (p < 0.001). The direction errors also remained significantly different after including co-stimulation magnitude as a nuisance variable (p < .001) though the magnitude errors were not significant afterwards (p = .054). We also repeated the mixed model analysis expressing the force prediction errors as a percentage of the co-stimulation force magnitude (Fig. 4b). The spinal/muscle errors were still significantly larger than muscle co-stimulation errors (p < 0.001). These results suggest that the larger errors observed for muscle-spinal co-stimulation were not due to larger magnitude responses.

**Figure 6:**
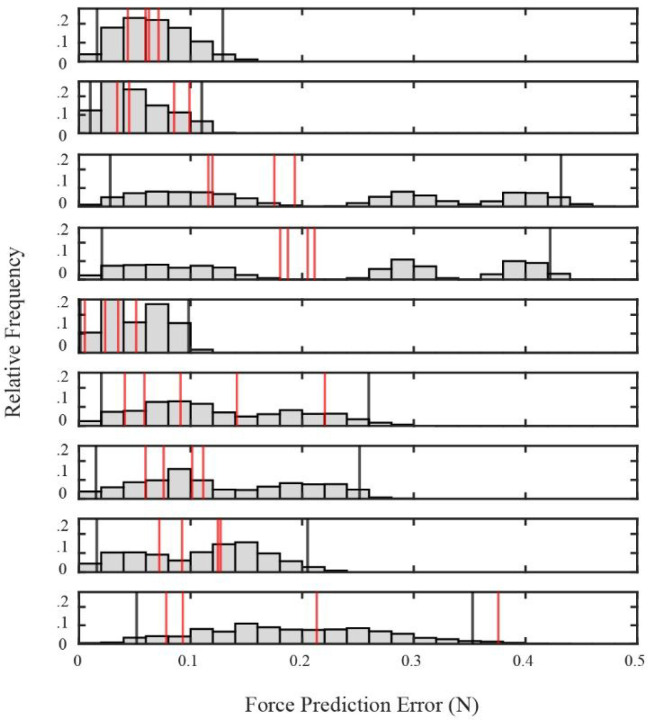
Estimated distributions of force prediction errors for nine unique pairs of co-stimulation amplitudes. The vertical red line indicates the actual force prediction errors from the observed data, most of which fall within the 95% confidence intervals (black vertical lines) of each distribution.

Larger prediction errors could also reflect differences in the variability of evoked responses: if the variability of spinal responses were higher than that of muscle responses, it could potentially lead to larger prediction errors in spinal/muscle co-stimulation trials. In fact, we found that the average standard deviations in the magnitude and direction of evoked forces for repeated trials of the same stimulation amplitude were considerably larger for spinal as compared to muscle stimulation (0.47N vs. 0.04N; 20.7° vs. 1.0°). To evaluate the possible contribution of this higher variability to the observed prediction errors, we performed a bootstrap analysis, estimating the distribution of prediction errors expected if spinal and muscle responses combined linearly but had the same variability as those observed for each response type. Figure 6 shows the expected distribution of prediction errors for unique pairs of spinal and muscle stimulation amplitudes, considering only those pairs for which enough repeated trials (n = 4) of each spinal and muscle amplitude in the pair were measured. For 8/9 pairs, the observed prediction errors (indicated by the red lines in each plot) were within the 95% confidence interval (indicated by black lines) of the estimated distribution of prediction errors. This result suggests that the larger prediction errors observed for spinal/muscle co-stimulation might primarily reflect the larger variability of spinal responses.

## Discussion

We found that forces produced by spinal/muscle co-stimulation were generally similar to the responses predicted by the linear sum of the forces from separate stimulation of each site. Prediction errors for these spinal/muscle co-stimulation trials, however, were larger than those from muscle/muscle co-stimulation. A subsequent bootstrap analysis found that these larger errors were likely due to the increased variability of spinal responses as compared to muscle responses. Taken together, these results provide limited evidence for non-linear interactions between the responses evoked between spinal and muscle stimulation. Our results have several implications as to the use of combined spinal and muscle stimulation for the restoration of movement following SCI.

### Nonlinear interactions between spinal and muscle stimulation

There were several reasons to expect a non-linear interaction between the effects of spinal and muscle stimulation. For example, antidromic motor unit potentials produced by the muscle stimulation could collide with orthodromic action potentials produced by spinal stimulation thereby resulting in a smaller than expected spinal/muscle stimulation output, as is the case for the M-wave in an H-reflex ^24^. Spinal cord mechanisms such as primary afferent depolarization ^25^ and persistent inward currents ^26^ might produce larger than expected motor outputs. Additionally, proprioceptive afferents could potentially be activated by muscle stimulation which might integrate with subthreshold activity evoked by spinal stimulation at motor neurons or spinal interneurons to generate nonlinearly summated motor outputs ^27–30^. On the other hand, other work has shown that simultaneous stimulation of different spinal sites is predicted by the linear combination of responses from each spinal site individually ^20–22,31^. Our results are more consistent with those experiments, in that we found limited evidence for non-linear interactions between responses from spinal and muscle stimulation. Future work could potentially investigate adjusting stimulation parameters such as muscle stimulation pulse widths to preferentially recruit sensory afferents ^32^ and inter pulse timing to expose any non-linear effects that may not have appeared in this study.

Our results suggest that the largest source of prediction error in the responses evoked by spinal and muscle stimulation is the trial-to-trial variability in the responses evoked from spinal stimulation. Such variability has been noted in previous work as well ^33,34^, and might be due to several different factors. It was recently shown that spikes traveling along proprioceptive afferents, which are the primary fibers activated by epidural spinal stimulation ^35^, may spontaneously fail to propagate down through the spinal cord ^25^. Time varying neuromodulation in the spinal cord may also contribute to response variability ^26,36^. The sudden increase in force magnitude with increasing currents in the spinal stimulation recruitment curves (Fig. 2) also suggests that spinal responses might be especially sensitive to small changes in neuromodulation or stimulation conditions. This latter possibility might be especially relevant in this study since we examined responses to S1 stimulation, which might be expected to activate a range of spinal roots^34,35,37,38^. It is possible that other spinal segments may differ in variability due to the anatomical arrangement of spinal roots on the dorsal aspect of the spinal cord ^35^. Other reasons for variability could be small changes in electrode positioning due to evoked movements, influences from supraspinal systems, depth of anesthesia, or fatigue. We also note that variability of evoked responses might be reduced after chronic SCI, due to the absence of supraspinal influences ^33,39^ or increased in awake behaving animals, due to increased neuromodulatory effects. Future work should consider these factors when applying a spinal/muscle stimulation strategy for rehabilitation.

### Implications for FES using Spinal/Muscle Stimulation Controllers

A spinal/muscle co-stimulation approach has the potential to enable flexible movement control after SCI. Despite having larger prediction errors than muscle co-stimulation, the error values we observe are not unreasonably large. More than half of the co-stimulation trials (66%) had force predictions errors lower than 30% of their co-stimulation force magnitudes, which is similar to relative force prediction errors for co-stimulation of many different muscles ^23^. As such, using the linear summation of individual inputs may be a reasonable estimate of the actual co-stimulation outputs. Prediction accuracies might be improved by collecting multiple spinal stimulation trials and incorporating spinal variability using other modeling techniques ^40,41^. Additionally, observed errors could also be used to estimate output uncertainty to improve the accuracy of controllers ^42^.

It is important to note, however, that translating a spinal/muscle co-stimulation approach into practical use will also require control of limb trajectories in addition to isometric forces, which do not consider the dynamic musculoskeletal properties of the limb and limb state dependent sensory feedback signals. Controllers using spinal/muscle co-stimulation will need to consider these factors to make accurate and useful predictions of limb movements that can ultimately be used in a rehabilitation context.

## Conclusion

In summary, we examined whether forces produced by spinal/muscle co-stimulation can be predicted by the linear summation of the forces produced by stimulation of each site separately. Although spinal/muscle co-stimulation outputs cannot be predicted as well as muscle co-stimulation, we find that accounting for the variability of spinal responses can explain this discrepancy. These results suggest that a spinal/muscle co-stimulation approach could be useful for a lower limb FES controller in patients with SCI.

## Methods

### Ethical Approvals

All procedures were approved by the Institutional Animal Care and Use committee at Northwestern University and performed in accordance with the relevant guidelines and regulations. All procedures are also in accordance with the essential 10 ARRIVE guidelines^43^.

### Surgical Procedure

A total of six female Sprague-Dawley rats (250–350 grams) were used in this study. Rats were anesthetized with an intraperitoneal injection of urethane (1.5 mg/kg). Urethane was used due to its relatively minimal effect on reflex responses as compared to other anesthetics ^44^. The animal’s level of anesthesia was monitored by the toe pinch response, respiratory rate, and whisker movements. Supplemental doses were administered as needed to achieve a deep anesthetic plane. The animal’s temperature was monitored using a rectal thermometer. Temperatures were kept between 36–38° C using a heating pad controlled by a DC temperature controller (40–90-8D, FHC Inc.).

Laminectomies were then performed at the L2 and L1 vertebrae to expose the spinal segments L4 through S1 ^45^. The exposed spinal cord was then covered and kept moist with a cotton ball soaked in saline for the remainder of the surgery. Incisions were then made to expose the illium and ischium contralateral to the stimulation site. A small hole was drilled into each site. Threaded rods (IMEX Veterinary Inc.) dipped in dental cement were then screwed into the holes and additional dental cement was applied to increase stability.

After the muscle electrodes were implanted (see below), the animal was then transferred to a raised platform. The pelvic posts were secured using an articulated holder (Noga Engineering). Lateral portions of L2 and L1 vertebra were dissected away to expose the transverse processes. Two clamps on either side of the spinal column were then used to stabilize the spinal column during stimulation. The ankle ipsilateral to the stimulation site was wrapped in a cuff and attached to a 6-axis force transducer (ATI-mini 40, ATI Industrial Automation Inc.). The force transducer was positioned such that the animal’s knee and hip joint were both approximately 90°. Upon completion of the experiment, animals were euthanized by intraperitoneal injection of Euthasol followed by a thoracotomy.

### Electrodes and Implantation

Spinal stimulation was performed with a Teflon coated 203 µm diameter solid silver electrode (786500, AM Systems Inc.). The stimulation electrode was initially positioned at the rostral border of the intact L3 vertebra. It was then moved 1 mm rostral and 500 µm laterally which approximately positioned it over the S1 spinal segment ^45,46^. Correct placement of the electrode was verified by observing right hindlimb movements in response to single pulse stimulation. The spinal cord was then bathed in mineral oil. Return electrodes (15 mm diameter stainless steel wire mesh) were placed subcutaneously on each side of the belly.

Muscle stimulation was performed using custom-made electrodes. Teflon coated multi-stranded wire (793200, A-M systems Inc.) was stripped to expose a 1 mm section of bare wire. To increase surface area and reduce current density, a stainless steel tube (23R304–1mm, Ziggy’s Tubes and Wires) was crimped over the exposed wire which was then filled with silver epoxy that was left to cure over several days. This resulted in a contact area of roughly 1 mm x 1 mm. For implantation, an incision was made over the lateral hindlimb parallel to the femur to expose the vastus lateralis (VL) and biceps femoris posterior (BF) muscles. Motor points were found by probing various locations on the surface of the muscle that generated the most complete visual muscle contraction in response to a 1 Hz suprathreshold stimulation using the electrode contact. The electrode contact was then threaded through the muscle near the motor point using a hook needle.

### Stimulation

Biphasic, charge balanced stimulation trains were delivered with an isolated battery powered stimulator (SI-8, Tucker Davis Technologies Inc.) to evoke hindlimb isometric contractions. A digital bioamp processor (RZ5, Tucker Davis Technologies Inc.) was used to synchronize force transducer data with stimulation timing. Spinal stimulation parameters were 15 ms period, 0.2 ms pulse width and 600 ms duration (40 pulses). Similar parameters were used to control hindlimb forces with spinal stimulation in previous work ^33^. Muscle stimulation parameters were 13 ms period, 0.12 ms pulse width and 520 ms duration (40 pulses). These parameters provided fused tetanic contractions ^23,47^. Current amplitude of both spinal and muscle stimulation was varied to control the amplitude of evoked responses.

A range of amplitudes were selected to identify the range of forces produced by each stimulation site (S1 spinal segment, VL and/or BF) and characterize recruitment curves. For each trial, the average baseline forces 400 ms prior to stimulation were subtracted to demean the force traces. Isometric forces were calculated using the average force in a 200 ms window beginning 300 ms after stimulation onset. After the recruitment curve was generated, one to three amplitudes for each spinal and muscle site were selected for co-stimulation trials. Using all combinations of the selected amplitudes, stimulation trials were then performed in blocks. The order of trials was randomized within each block. Each trial was separated by at least 45 seconds of rest. Table 1 outlines an example randomized stimulation block.

### Linearity Analysis

Analyses were performed using custom scripts in MATLAB Version 2018b (Mathworks, Natick, MA). Circular statistics were performed using the CircStat Toolbox^48^. For each co-stimulation trial of two sites at specific current magnitudes, we compared the observed force vector to a prediction vector. The prediction vector was calculated from the sum of the vectors evoked from stimulation of each site individually, using the responses collected within the same block as the co-stimulation trial. Force prediction errors were calculated as the Euclidean distance between the prediction vector and observed co-stimulation vector. Magnitude errors were calculated as the difference between the magnitudes of the observed and predicted co-stimulation vectors. Direction errors were calculated as the angle between predicted and observed vectors. Only trials producing force magnitudes greater than 0.03 N were included for subsequent analyses to ensure evoked force vectors were well defined above background noise.

We compared the errors found by co-stimulation of an epidural spinal site and a muscle site to the errors found by co-stimulation of two different muscles (VL, BF) within each animal. Responses evoked from stimulation of two muscles have been observed to combine linearly, especially for muscles well separated in the limb such as VL and BF used here ^23^. If the errors observed for combined spinal/muscle stimulation were larger than those observed for combined muscle/muscle stimulation, it would suggest that spinal and muscle co-stimulation vectors cannot be predicted by linear summation. We used linear mixed models to assess whether errors differed significantly between the two types of co-stimulation trials. A separate model was fit for each of the three errors (force prediction, magnitude, and direction). Errors were log transformed as necessary to satisfy the model’s assumption of normal distribution of residuals. Co-stimulation type (spinal/muscle vs muscle/muscle) was set as a fixed factor. Subject was set as a random factor with randomly varying slope and intercept. The force magnitude evoked by co-stimulation was also included as a fixed factor since larger co-stimulation force magnitudes could potentially correlate with larger force prediction errors ^23^. A maximum log likelihood estimator was used to fit model parameters.

### Bootstrapping Analysis

As described below, the responses from spinal stimulation were often variable across repeated trials and this variability was often larger than that of repeated muscle stimulation. It was therefore possible that larger prediction errors for spinal/muscle co-stimulation trials as compared to muscle/muscle co-stimulation trials could reflect the variability of responses rather than any non-linear interactions between spinal and muscle stimulation. We therefore performed a bootstrap analysis to evaluate the effects of response variability on observed force prediction errors.

We considered cases of unique pairs of spinal/muscle co-stimulation current amplitudes that were repeated at least 4 times to evaluate the trial-to-trial variability of individual spinal and muscle responses. We excluded cases that had a significant (p < .01) decrease in force magnitude over the duration of the experiment, as determined by a linear regression with spinal force magnitude as the dependent variable and time (in minutes) as the dependent variable. With these inclusion criteria, we identified 9 cases which could be used to evaluate the effects of variability on force prediction errors.

For each case we generated 5000 trials of spinal and 5000 trials of muscle responses by resampling the observed data with replacement. The trials in each sample were then linearly summed to generate 5000 trials of simulated ‘observed’ co-stimulation trials; these trials represent the co-stimulation vectors expected if spinal and muscle responses combined linearly and which had the variability of the observed responses. This process was then again repeated to generate 5000 new trials of spinal and muscle responses each. These separate spinal and muscle responses were then used to generate a ‘predicted’ force vector expected if the individual responses combined linearly. By calculating the difference between simulated observed and predicted vectors, we generated a distribution of 5000 force prediction errors. The distribution was then used to calculate 95% confidence intervals. The actual mean force prediction errors from our observed samples (i.e. from the real data) was then compared to the confidence interval. If the actual error was likely with respect to the distribution (i.e. within the 95% confidence interval), it would suggest that the observed errors reflected the trial-to-trial variability of the spinal responses rather than any non-linear interactions between spinal and muscle responses.

## Figures and Tables

**Table 1:** Example block of trials performed for two spinal and two muscle inputs

Trial #	Muscle Input Current	Spinal Input Current
1	1333 µA	233 µA
2	0 µA	272 µA
3	1000 µA	0 µA
4	1333 µA	0 µA
5	1333 µA	272 µA
6	0 µA	233 µA
7	1000 µA	272 µA
8	1000 µA	233 µA
